# Clustering of noncommunicable disease risk factors among adolescents attending higher secondary schools in Kasaragod District, Kerala, India [version 2; peer review: 1 approved with reservations]

**DOI:** 10.12688/wellcomeopenres.16873.2

**Published:** 2021-09-23

**Authors:** Thekke Veedu Sreena, Elezebeth Mathews, Prakash Babu Kodali, Kavumpurathu Raman Thankappan

**Affiliations:** Department of Public Health and Community Medicine, Central University of Kerala, Kasargod, Kerala, 671320, India

**Keywords:** NCD risk factors, clustering, adolescents, Kasaragod, Kerala, India

## Abstract

**Background:**

Limited evidence exists on the presence of collective non-communicable disease (NCD) risk factors among adolescents in Kerala, India. We aimed to assess the prevalence and factors associated with multiple NCD risk factors and the clustering of these risk factors among adolescents in Kasaragod District, Kerala.

**Methods:**

We selected 470 adolescents (mean age 16.6 years, male 53.8%) through multi-stage cluster sampling from higher secondary schools of Kasaragod district. Self-administered questionnaires were used, and anthropometric measurements were taken using standard techniques and protocols. Tobacco use, alcohol consumption, low fruits and vegetable consumption, inadequate physical activity, extra salt intake, overweight, consumption of soft drinks and packed foods were the eight NCD risk factors included.The factors associated with one, two and three or more NCD risk factors were analysed using multinomial logistic regression and the standard errors were adjusted for the four clusters.

**Results:**

Risk factor clusters with two risk factors (dyads) and three risk factors (triads) were observed in 163 (34.7%) and 102 (21.7%) of the sample, respectively. Adolescents residing in urban areas (odds ratio (OR) = 3.55; 95% confidence interval (CI) = 1.45-8.73), whose father’s education level was lower (OR = 3.54; 95% CI = 1.24-10.10), whose mother’s education was lower (OR= 4.13; 95% CI = 1.27-13.51), who had restrictions on physical activity (OR = 5.41; 95% CI = 1.20-24.30) and who did not have a kitchen garden (an area where fruits and vegetables are grown for domestic use) (OR=4.51;95% CI = 1.44-14.12) were more likely to have three or more NCD risk factors compared to their counterparts.

**Conclusions:**

Clustering of NCD risk factors was prevalent in more than half of the adolescents. Efforts are warranted to reduce multiple risk factors, focussing on children of low educated parents and urban residents.

## Introduction

Non-communicable diseases (NCDs) such as cardiovascular diseases, cancer, diabetes, chronic respiratory diseases are the leading cause of death globally. According to the World Health Organization (WHO), NCDs contribute to 71% (41 million) of all deaths globally and 60% (5.87 million) of all deaths nationally^1^. About 4% of all NCD deaths in the year 2016 occurred in people below 30 years^2^. Exposure to NCD risk factors starts from the womb itself and aggravates in the later years of life. Prenatal exposure to tobacco and alcohol, maternal diabetes, overnutrition *in utero,* intrauterine growth retardation, premature birth, nutritional deficiency, and intergenerational factors have long-term impacts on health, including increased risk of adult cardiovascular disease, diabetes, etc^3^. NCD prevention is of utmost importance as it affects individuals in the productive life years and is a challenge to sustainable human development. Although lifestyle modification is an effective strategy to prevent and reduce the burden of NCDs^4^, their scope in prevention is limited in the adult stage. Compelling evidence suggests that the onset of NCDs starts in the early years of development and any intervention to combat the disease should be targeted in the early years of life^5^. One of the key actions to curtail the incidence of NCDs is to prevent and control NCD risk behaviours among the adolescent population. The adolescence stage shapes the nutritional, physical activity, and other lifestyle behaviours that are mostly taken forward to adulthood^6^. As per the Global Adult Tobacco Survey (GATS)-2, adolescents in the age group 15–19 years contribute to over 30% of tobacco users among youth in the age 15–24 years^7^. Within India, Kerala is the most advanced state in epidemiological transition (Lancet nations within a nation) and has a high prevalence of behavioural and metabolic risk factors of NCD^8^. According to the latest NCD risk factor survey in the state using the World Health Organization (WHO) stepwise approach to surveillance (STEPs) approach, the state had a diabetes prevalence of 19.2% and a hypertension prevalence of 30% in the adult population of the state^9^. Abdominal obesity was 60% (men 39%, women 73%)^9^. Studies conducted among adolescents reported the presence of soaring prevalence of certain NCDs risk factors globally and in India.

The need to target adolescents to prevent the development of NCD risk factors is globally recognized. “Catch them young and keep them healthy” has become a catchphrase in global NCD prevention^10^. Though it is known that the collective impact of multiple risk factors could increase NCD risk, limited studies captured the presence of collective NCD risk factors and clustering of these risk factors among adolescents in Kerala. Given the context, this study was conducted with an objective to assess the prevalence of multiple NCD risk factors and clustering of these risk factors among adolescents in the Kasaragod District of Kerala and to find out the factors associated with the multiple risk factors among adolescents.

## Methods

### Study design

The study was conducted using multi-stage stratified cluster sampling method in February 2018. Amongst the two educational districts in Kasaragod district, one was selected randomly. This study targeted only higher secondary students i.e., from the age group of 15 – 19 years (late adolescent stage) from both public and private schools. Keeping in view of the anticipated prevalence of 5.2%^11^, confidence interval of 95%, precision factor of 3%, a design effect of two and non-response rate of 10%, a total sample size of 470 was estimated. Those who were physically absent during the data collection were excluded from the study.

A self-administered structured questionnaire was used for data collection^12^. The questionnaire captured socio-demographic characteristics, known predictors of behavioural risk factors and NCDs such as family history of obesity, body image perception, physical activity behaviour and factor influencing it, exposure to tobacco, alcohol use among parents and dietary habits. In addition to this, the Indian Adolescent Health questionnaire (IAHQ), validated in India^13^ and B.G Prasad scales^14^ were used to measure the outcome variables. The modified questionnaire was piloted in a smaller sample of 15 students for the feasibility and comprehension of the questions among the students, who were excluded in the final survey. Minor rephrasing of the questions was made as per the feedback and the questionnaire was found to be comprehendible and feasible to be employed and was finalized thereafter.

All behavioural risk factors were based on the Indian Adolescent Health Questionnaire. Physical activity was defined as any activity that increases the heart rate and makes them get out of breath some of the time. If the participants were physically active for at least 30 minutes per day for five days in the previous week of the survey they were considered as physically active. Tobacco use was defined as the use of any form of tobacco products (smoking and smokeless) in the previous month. Current alcohol use was defined as use of any alcoholic products such as wine, vodka, beer, or whiskey, etc in the last six months except drinking few sips of wine for a religious purpose. Ever user was defined as one who used tobacco products/ consumed alcohol at least once in their life-time. Inadequate intake of fruits and vegetables was defined as consuming less than five servings of fruits and vegetables per day. Anthropometric measurements such as height in centimetres and weight in kilogram were taken at school on the day of the survey using standardized instruments like SECA wall-mounted measuring scale and SECA electronic weighing scale using standard protocol^15^. Body mass index (BMI) for age was computed using WHO growth reference for school aged children and adolescents^16^. Adolescents were categorized overweight if BMI for age was greater than one standard deviation from median^16,17^.

### Ethical approval

Ethical clearance was obtained from the Institutional Human Ethics Committee (IHEC) of the Central University of Kerala (CUK/IHEC/2018/018, 19 February 2018). Authorization for data collection was taken from the higher secondary district coordinator and the respective school heads. Students who met the inclusion criteria, were briefed about the study and provided with participant information sheet and informed consent, a day prior to the data collection. Participants were recruited only after obtaining consent from parents and assent from the students. The survey and anthropometric measurements were taken on the subsequent day at the school. Anthropometric measurements were taken after ensuring sufficient privacy to the participants. Confidentiality of the individuals was ensured by masking the personal identifiers with a participant identification number.

### Data analysis

The data were entered, cleaned and analysed using SPSS version 23.0. Frequencies, proportions and percentages were used to descriptively analyse the data. Prevalence of individual risk factors were analysed. Descriptive analysis of the clustering of NCD risk factors were done. The factors associated with one, two and three or more NCD risk factors were analysed using multinomial logistic regression and the standard errors were adjusted for the four clusters. The independent variables that were potential confounders and effect modifiers such as age, education, gender and the behavioural risk factors were included in the model.

## Results

A sample of 470 adolescents in the ages 16–19 years were analysed^12^. The sample comprised of 53.8% (n=253) males and 46.2% (n=217) females. The mean age (in completed years) of the participants was 16.6 years. The majority of the participants was from urban areas (66.4%). A detailed outline of the socio-demographic characteristics of the sample are given in Table 1.

### Prevalence of NCD risk factors

NCD risk factors were highly prevalent among the study sample. This study assessed the prevalence of eight major NCD risk factors. Among the NCD risk factors, consumption of packed food was most prevalent, with 67% adolescents reporting consumption of packaged food in the last one week. Prevalence of inadequate fruit and vegetable intake was 49%, followed by physical inactivity (41.9%). The least prevalent risk factor was tobacco use (smoke form or smokeless form) of 4.7% (n=22). The detailed outline of the eight risk factors such as fruit and vegetable intake, consumption of soft drinks, overweight, physical inactivity, alcohol consumption, tobacco use, extra salt intake and consumption of packed food is given in Table 2.

### Clustering of NCD risk factors

**Figure 1:**
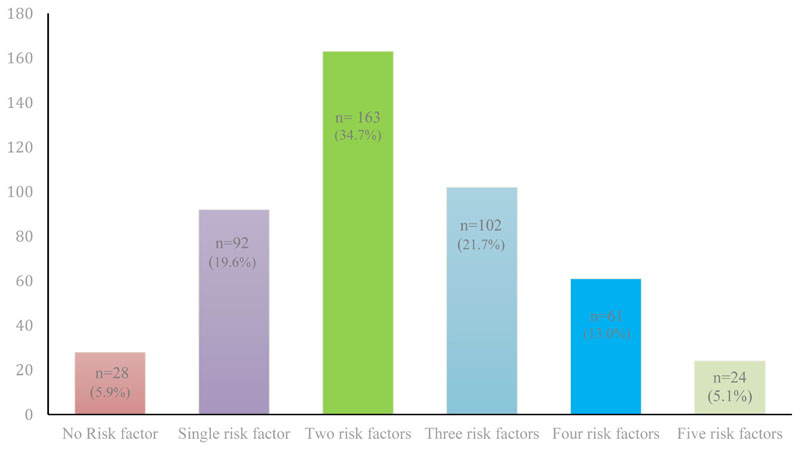
Profile of non-communicable disease (NCD) risk factor clusters in the sample (n=470).

Among 470 adolescents sampled, at least one of the eight NCD risk factors were observed in 94.1% (n= 442) of the sample. Interestingly, the NCD risk factors were found to be clustering with each other in most of the sample. Five of the eight NCD risk factors were reported by 5.1% (n=24) of the total sample. Risk factor clusters with two risk factors (dyads) and three risk factors (triads) were observed in 163 (34.7%) and 102 (21.7%) of the sample respectively. Overall, 39.8% of the total sample were found to have at least three NCD risk factors. More than five risk factors were not reported by anyone. Figure 1 represents the NCD risk factor profile of the sample.

**Figure 2:**
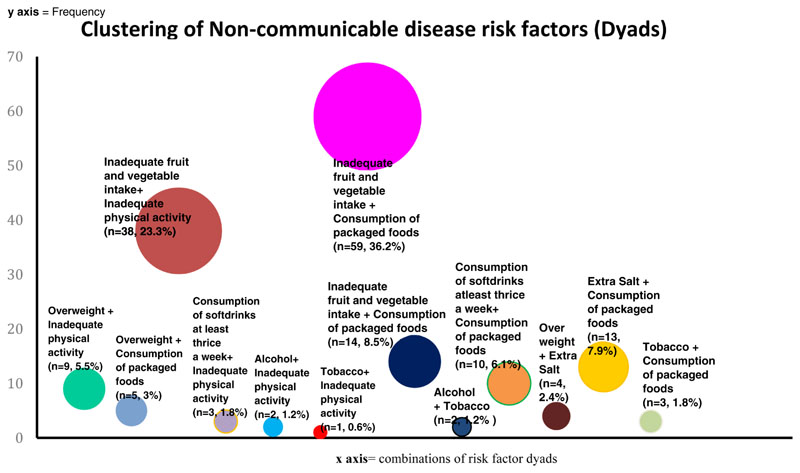
Clusters (Dyads) of non-communicable disease (NCD) risk-factors among adolescents (n= 163).

**Figure 3:**
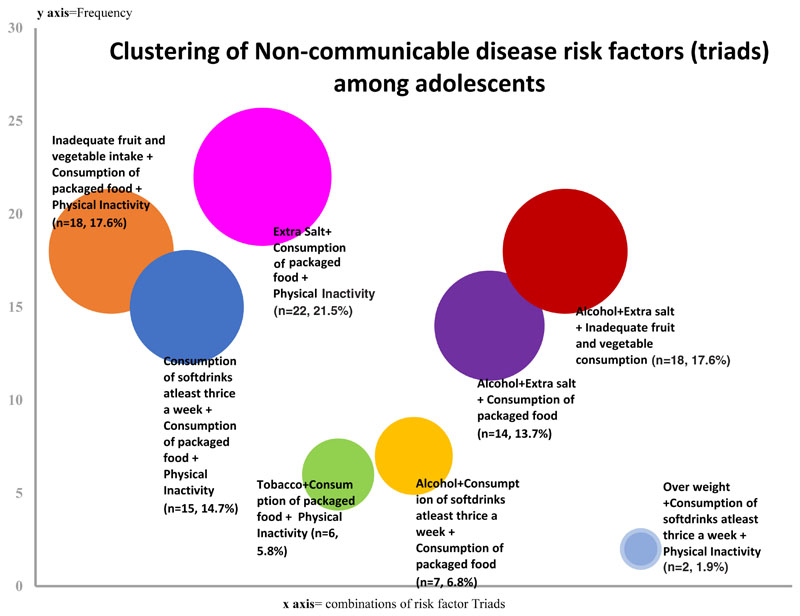
Clusters (triads) of non-communicable disease (NCD) risk-factors among adolescents (n=102).

Dyads and triads of NCD risk factors were highly prevalent among the adolescents accounting for a combined prevalence of 56.4% (n=265). We decomposed the dyads and triads to identify the NCD risk factor combinations. Among the dyads, the combination of “inadequate fruit and vegetable intake + consumption of packaged food” were most prevalent. Among the triads, the combination of “extra salt + consumption of packaged food + physical inactivity” were most prevalent. Figure 2 and Figure 3 report the decomposition of NCD risk factor cluster dyads and triads, respectively.

### Factors associated with clustering of NCD risk factors

Unadjusted odds ratios were computed to identify the factors associated with clustering of NCD risk factors among the adolescents. Factors found to have significant unadjusted odds were included into multi-variate analysis. Multivariate analysis was conducted using multinomial logistic regression with NCD risk factors (i.e., one risk factor, two risk factors, three or more risk factors) as dependent variable. The multinomial logistic regression yielded a significant model with an acceptable model fit. Several predictive factors such as gender of the participant, place of living, educational status of mother, father’s education, restrictions on physical activity, and having an income generating job were found to be significantly predicting the clustering of NCD risk factors. The resultant adjusted odds ratios with 95% confidence intervals obtained through multinomial logistic regression are outlined in Table 3.

Factors associated with the presence of at least two NCD risk factors were female gender (OR = 5.51, 95% CI = 3.35-9.07), urban residence (OR = 2.79, 95% CI =2.06-3.78), adolescents father’s low education level (OR = 4.39, 95% CI = 2.07-9.28), adolescents mother’s low education level (OR= 7.55 (3.19-17.89) and the adolescent having an income generating job (OR = 6.07, 95% = 2.39-1.4). NCD risk factor clusters with three or more risk factors were associated with female gender (OR= 1.56, 95% CI= 1.04-2.32), urban residence (OR = 3.55, 95% CI = 1.57-7.99), adolescents father’s low education level (OR = 3.54, 95% CI = 2.15-5.80), restrictions on physical activity (OR = 5.40, 95% CI = 2.83-10.32) and having kitchen garden in home (OR=4.50, 95% CI = 1.28-15.78))

## Discussion

The study was conducted to assess the prevalence of NCD risk factors among adolescents and identify the clustering of risk factors and their correlates. Our study found that NCD risk factors among the adolescents were majorly on unhealthy diet and physical inactivity. High prevalence of these risk factors among Indian adolescents were documented in earlier studies. A study comparing NCD risk factors among adolescents in five southeast Asian countries observed that over 85% of the adolescents in India had inadequate fruit and vegetable consumption (i.e., < 5 servings per day)^18^. While limited evidence exists concerning the consumption of packaged food among adolescents in Indian context, studies from other developing countries argue that affordability of packaged foods, peer influence, absence of healthy alternatives and perception of packaged foods as safer options make them popular food choice among adolescents^19^.

Interestingly, the study observed the clustering of the NCD risk factors among the adolescents. Among the risk factor dyads, “Inadequate fruit and vegetable intake along with consumption of packaged foods” was the most prominent followed only by the dyad of “inadequate fruit and vegetable intake plus inadequate physical activity”. Among NCD risk factor triads “extra salt + consumption of packaged food + physical inactivity”, “inadequate fruit and vegetable intake + consumption of packaged food + physical inactivity” and “alcohol+ extra salt + inadequate fruit and vegetable consumption” were among the major ones. A recent study from north India reported that physical inactivity and inadequate fruit and vegetable intake make up the largest of the behavioural risk factor dyads among adolescents^20^. While in our study inadequate fruit and vegetable intake, physical inactivity and consumption of packaged food were found to be strong contributors to NCD risk factor clusters, earlier studies reported obesity and overweight as predominant risk factors in NCD clusters among adolescents in north India^21^. Physical inactivity along with consumption of packaged food during adolescent period is likely to contribute to overweight while these adolescents become adults. Several factors were found to be significantly predicting the NCD risk factor clustering among adolescents. Being a female, living in urban area, father having an education of up to higher secondary schooling, mother’s education of graduation and above, and possessing an income generating job were found to be significantly predicting the clustering of NCD risk factor dyads among the adolescents. In the study it was found that females had higher odds (OR- 5.51) of having at least two NCD risk factors (dyads) compared to males. This is in contrast to a recent study from north India which reported a higher prevalence of NCD risk factor dyads among males compared to females^20^. Similarly, adolescents from urban regions had higher odds of NCD risk factors and their clustering compared to rural counter parts. It could primarily be due to the reason that adolescents in urban region have better transport facilities, fewer possibilities to undertake physical activity, easy availability of unhealthy foods, and other risk factors. The higher prevalence of NCDs and NCD risk factors among urban adults is well known^22^. An interesting observation made with respect to NCD risk factor clustering was with regard to restriction in physical activity. Adolescents who reported restriction in physical activity had a high odds (OR = 5.40, 95% CI = 2.83-10.32) of developing NCD risk factor clusters with three or more NCD risk factors. Literature from other developing country settings reported that parenting practices influence development of NCD risk factors among adolescents^23^. While restrictions on physical activity prevent the development of certain peer influenced NCD risk factors such as tobacco and alcohol, it can substantially increase the chances for NCD risk factors of physical inactivity, overweight, consumption of packaged food etc. Tobacco use was the least prevalent risk factor among this population. Tobacco consumption in most Indian states reduced as per the Global Adult Tobacco Survey 2 and Kerala reported the highest reduction of tobacco use among the major Indian states^7^. Therefore, this finding is in line with the findings of the GATS survey.

One of the limitations of the study is that it surveyed adolescents attending an educational institution and hence may not be representative of the community Behavioural risk factors such as physical activity, diet, alcohol and tobacco use were self-reports may likely have reporting bias.

In conclusion, there was high prevalence of individual NCD risk factors and risk factor clusters among the adolescents in Kasaragod, Kerala. Most NCD risk factors were dietary in nature, specifically around consumption of packaged food or inadequate consumption of fruits and vegetables. Indian policy environment gives a lesser emphasis to encourage healthy eating among adolescents compared to its LMIC counter parts^24^. There is a need to prioritize healthy eating by the governments, education department and schools. Moreover, targeted interventions should also focus on improving physical activity and preventing the initiation of alcohol and tobacco use.

## Supplementary Material

Supplementary Material

## Figures and Tables

**Table 1:** Socio-demographic profile of the participants (n=470) Variables.

Variables	Frequency	Percentage
Gender		
Male	253	53.8
Female	217	46.2
Place of living		
Urban	312	66.4
Rural	158	33.6
Religion		
Hindu	330	70.2
Muslim	060	12.8
Christian	080	17.0
Type of family		
Nuclear family	442	94.0
Joint family	028	06.0
Living with		
Both parents	393	83.6
Mother only	074	15.7
Family history of Obesity		
Yes	385	82.0
No	85	18.0
Engaged in any income generating job		
Yes	054	11.5
No	416	88.5

**Table 2:** Non-communicable disease (NCD) risk factors among the adolescents (N=470).

Risk factor	Frequency (n)	Percentage (%)
*Fruit and vegetable intake*		
Adequate	238	50.6
Inadequate	232	49.4
*Consumption of soft drinks*		
Up to two times per week	424	90.2
At least three times per week	46	9.8
*Overweight*		
Yes	62	13.2
No	408	86.8
*Physical inactivity*		
Yes	197	41.9
No	273	58.1
*Alcohol consumption*		
Yes	91	19.4
No	379	80.6
*Tobacco use*		
Yes	22	4.7
No	448	95.3
*Extra salt use*		
Yes	123	26.2
No	347	73.8
*Consumption of packed food in the last one week*		
Yes	315	67.0
No	155	33.0

**Table 3:** Predictors of non-communicable disease (NCD) risk-factors among adolescents: Results of multinomial logistic regression. (CI=confidence interval, Standard errors adjusted for the four clusters).

	One risk factor Odds Ratio (95% CI)	Two risk factors Odds Ratio (95% CI)	Three or more risk factors Odds Ratio (95% CI)
***Gender***			
Female	**3.07 (2.28-4.15)**	**5.51(3.35-**9.07)	**1.56 (1.04-2.32)**
Male	Ref	Ref	Ref
***Place of living***			
Urban	3.07 (1.14**-8.27**)	**2.79 (2.06-3.78)**	**3.55 (1.57-7.99)**
Rural	Ref	Ref	Ref
***Age***			
Up to 16 years	2.16 (1.94-2.41)	0.79 (0.48-1.29)	**1.48(1.03-2.14)**
17 years and above	Ref	Ref	Ref
***Father's education***			
Up to higher secondary	2.01 (0.71-5.69)	**4.39 (2.07-9.28)**	** 3.54 (2.15-5.80)**
Graduation and above	Ref	Ref	Ref
***Mother's education***			
Graduation and above	2.81 (0.72-10.93)	**7.55 (3.19- 17.89)**	**4.13 (1.86-9.17)**
Up to higher secondary	Ref	Ref	Ref
***Restrictions on Physical activity***			
Yes	0.**08** (0.01-**0.67**)	1.58 (0.51-4.92)	**5.40 (2.83-10.32)**
No	Ref	Ref	Ref
***Kitchen garden in home***			
No	2.46 (0.50-11.9)	0.52 (0.13-2.05)	**4.50 (1.28-15.78)**
Yes	Ref	Ref	Ref
***Family history of obesity***			
Yes	1.09 (0.45-2.63)	0.26 (0.13-0.51)	0.68 (0.26-1.74)
No	Ref	Ref	Ref
***Has an income generating job***			
Yes	3.83 (2.72-4.4)	6.07 (2.39 -15.4)	1.03 (0.62-1.72)
No	Ref	Ref	Ref

## Data Availability

Open Science Framework: NCD risk factors among adolescents. https://doi.org/10.17605/OSF.IO/H4RPG^12^. This project contains the following underlying data: - NCD Risk factors among Adolescents.csv (The dataset includes socio-demographic information, eight non-communicable disease risk factors and anthropometric measurements such as height and weight of school going adolescents aged 15–19 years) Data are available under the terms of the Creative Commons Attribution 4.0 International license (CC-BY 4.0). Open Science Framework: NCD risk factors among adolescents. https://doi.org/10.17605/OSF.IO/H4RPG^12^. This project contains the following extended data: - Informed assent form.pdf - Informed consent (English).pdf - Participant information sheet – parents.pdf - Participant information sheet – students.pdf - Study questionnaire.pdf - Codebook.csv (Code book for the data set)

## Amendments from Version 1

In the revised version of the manuscript, the factors associated with one, two and three or more NCD risk factors were analysed using multinomial logistic regression and the standard errors were adjusted for the four clusters. The independent variables that were potential confounders and effect modifiers such as age, education, gender and behavioural risk factors were included in the model.
