# Supplementary material for: Clustering of noncommunicable disease risk factors among adolescents attending higher secondary schools in Kasaragod District, Kerala, India [version 2; peer review: 1 approved with reservations]
Source: Wellcome Open Res. Author manuscript; Available in PMC 2022 Jun 10. (PMC7612837; doi:10.12688/wellcomeopenres.16873.2)
Supplement: Supplementary Material [file EMS145408-supplement-Supplementary_Material.pdf]

Open Peer Review

Current Peer Review Status: ?

Version 1

Reviewer Report 12 August 2021

https://doi.org/10.21956/wellcomeopenres.18612.r45163

© 2021 Lim K. This is an open access peer review report distributed under the terms of the Creative Commons Attribution License, which permits unrestricted use, distribution, and reproduction in any medium, provided the original work is properly cited.

? **Kuang H. Lim**
Special Resource Centre, Institute for Medical Research, Kuala Lumpur, Malaysia

The manuscript on Clustering of noncommunicable disease risk factors among adolescents attending higher secondary schools in Kasaragod District, Kerala, India, is of public health important and well written, in which the authors have justified the study by stated the problem and burden of Non-communicable diseases globally and in India. In addition, the authors stated that all health risk behaviour usually "started" during adolescence and limited information required the study to be carried out in Kerala. The authors clearly stated how the sampling was carried out and the valid tool was used for data collection.

However, there are some lacking aspects of the analysis the results, in which the authors only categorised the clustering based on the descriptive aspect, while there are statistic methods such as clustering analysis which can further enhance the quality of the analysis. In addition, the authors did not describe on the fit of the multinomial logistic model, in which several assumption need to be fulfilled to ensure the results of the study are valid. (such as linearity, no outlier etc). In addition, the possible two way interaction between the independent variables (IV) need to be tested to ensure no "Effect Modification" among IV.

The results are presented in systematic way and discussion is quite comprehensive (based on the results in the text). But this can be improved by expanding it to the findings from more developed and developing countries, (consistent and contrast and why) and not limited it on the plausible reasons for the findings in this study.

Is the work clearly and accurately presented and does it cite the current literature?
Yes

Is the study design appropriate and is the work technically sound?
Yes

Are sufficient details of methods and analysis provided to allow replication by others?

Partly

If applicable, is the statistical analysis and its interpretation appropriate?
Partly

Are all the source data underlying the results available to ensure full reproducibility?
Yes

Are the conclusions drawn adequately supported by the results?
Yes

Competing Interests: No competing interests were disclosed.

Reviewer Expertise: adolescent health, smoking, non-communicable diseases

I confirm that I have read this submission and believe that I have an appropriate level of expertise to confirm that it is of an acceptable scientific standard, however I have significant reservations, as outlined above.
